# Silencing of SNHG6 induced cell autophagy by targeting miR-26a-5p/ULK1 signaling pathway in human osteosarcoma

**DOI:** 10.1186/s12935-019-0794-1

**Published:** 2019-04-03

**Authors:** Xin Zhu, Guangling Yang, Jisheng Xu, Chuanlin Zhang

**Affiliations:** Department of Orthopedics, Shangqiu First People’s Hospital, No.292, Kaixuan Road, Shangqiu, 476100 China

**Keywords:** SNHG6, Osteosarcoma, Poor clinical prognosis, miR-26a-5p, ULK1, Apoptosis, Autophagy

## Abstract

**Background:**

lncRNAs have been proved to play crucial parts in various human cytopathology and cell physiology, including tumorigenesis. Down-regulated lncRNAs SNHG6 have shown great cell proliferation inhibitory effects in cancer development. Here we investigated how SNHG6 effected human osteosarcoma (OS) development and progression. Methods: Reverse transcription-quantitative PCR was performed to detect SNHG6 mRNA level in both OS tissues and cell lines. MTT and colony formation assays were used to determine the growth impact of SNHG6. Wound healing and trans-well assay were performed to measure the invasion effect of SNHG6. Western blotting were utilized to dissect molecular mechanisms.

**Results:**

We identified SNHG6 as a lncRNAs that significantly up-regulated in OS tissues and cells, patients with high SNHG6 expression suffered more malignant metastasis and shorter survival times. Furthermore, silencing of SNHG6 in OS significantly inhibited OS cell growth, weakened cell invasion capacity, arrested cell cycle at G0/G1 phase, and induced cell apoptosis. Additionally, mechanism assays suggested that SNHG6 could competitively sponging miR-26a-5p thereby regulating ULK1, and induced cell apoptosis and autophagy by targeting caspase3 and ATF3. Conclusions: Our findings demonstrated that SNHG6 acted as an oncogene in osteosarcoma cells through regulating miR-26a-5p/ULK1 at a post-transcriptional level. SNHG6 might serve as a candidate prognostic biomarker and a target for novel therapies of osteosarcoma patients.

## Background

Osteosarcoma (OS), which remains the most frequent human primary solid malignancy bone cancer, has been suggested to be a differentiation disease caused by malignant mesenchymal cells differentiation interrupt [1, 2]. OS mainly presents in children and young adult at 15–19 years of age, accounting for almost 60% of malignant bone tumors, moreover, the incidence of OS was about 8–11/million/year throughout the world [3, 4]. OS has highly aggressive and early metastatic potential [5]. Approximately 10%–25% of patients occur lung metastasis and die of pulmonary metastasis within 1 year [6]. The use of effective adjuvant chemotherapies plays an important role in controlling OS. Combination of chemotherapeutics and surgery resection have been used for human OS treatment, which significantly increased OS patients’ survival ratefrom 20% at 1979s to 65%–75% at 2012, however less than 30% patients with metastasis disease can survive [7–10]. Thus, it was necessary to develop more effective adjuvant treatments.

Long non-coding RNAs (lncRNAs) were numerous transcripts that consist of over 200 nucleotides. Though 90% human genome DNA were widely transcribed, 98% of them encode none protein, which were identified as non-coding RNAs (ncRNAs) [11, 12]. LncRNAs make up more than 70% of ncRNAs, [13] and they have received more and more attention for the new function in tumorigenesis, metastasis and chemoresistance [14]. Therefore, more researchers were seeking for lncRNAs which might contribute to the pathogenesis or chemoresistance of OS.

During our research, we found that nucleolar RNA host gene 6 (SNHG6) was over-expressed in primary tumor samples and OS cell lines (SOSP-9607 and MG63). Furthermore, SNHG6 was identified as a tumor promoter targeting miR-26a-5p, as reduction of SNHG6 expression in OS cell lines was able to reduce cell viability and migration in vitro, arrest cell cycle at G0/G1 phase, induce cell apoptosis. Additionally, unc-51 like autophagy activating kinase (ULK1), a key component of autophagy pathway, was identified to be a novel direct target of miR-26a-5p. And mechanism experiments uncovered that SNHG6 might functioned through sponging miR-26a-5p and thus regulating cell apoptosis and cell autophagy by targeting ULK1. Collectedly, we might find a novel therapy strategy based on lncRNA-directed against human OS.

## Methods

### Patients and tumor samples

Osteosarcoma tumor tissues and adjacent normal tissues were collected from 30 primary osteosarcoma patients among 2006 and 2009 at Shangqiu First People’s Hospital. Following surgery, tissues samples were stored at liquid nitrogen until subsequent experiments.

Informed consents were obtained from all individual patients involved in this research. All procedures performed in studies involving human participants were in accordance with the ethical standards of the Ethics Review Committee of Shangqiu First People’s Hospital (Shangqiu, China). The characteristics of these patients were shown in Table 1.

**Table 1:** Correlation between the expression of SNHG6 and clinical characteristics of OS

Factor	Number	SNHG6 expression		*P*
		Low	High	
Age (years)				0.12
< 60	11	7	4	
≥ 60	19	9	10	
Gender				0.12
Male	18	7	11	
Female	12	8	4	
Histological grade				0.49
Low or undiffer	17	8	9	
Middle or high	13	5	8	
Tumor invasion depth				0.001*
T1	13	8	5	
≥ T2	17	4	13	
Lymph node metastasis				0.011*
N0	13	9	4	
≥ N1	17	6	11	
Distant metastasis				
M0	24	12	12	0.75
M1	6	2	4	
TNM stage				0.001*
I–II	12	7	5	
III–IV	18	5	13	

* P < 0.05

### Cell culture

Human normal osteoblastic cell line hFOB, human osteosarcoma cell lines SOSP-9607 and MG63 cells were obtained from American Type Culture Collection (ATCC). SOSP-9607 cells was maintained in RPMI 1640 medium (HyClone), 2.0 mM l-glutamine, 100 U/ml penicillin, and 100 ug/ml streptomycin, while other cells were maintained in the same conditions, except that Dulbecco’s modified Eagle’s medium (DMEM, Sigma) was used. All the media were supplemented with 10% dialyzed fetal bovine serum (FBS; HyClone) at 37 °C in a humidified incubator containing 5% CO_2_.

### Cell transfection

hFOB, SOSP-9607 and MG63 cells were seeded into each well of 6-well plate of 5 × 10^3^ cells, respectively. Then, transfected with human SNHG6 small interfering RNA (siRNAs) (Cat. No.: iV022927) or negative control (NC) siRNA, which were purchased from Applied Biological Materials Inc. (abm) and the corresponding sequences were as follows: siRNA-SNHG6, CGCGAAGAGCCGTTAGTCATGCCGGTGTG; siRNA-NC, UUCUC CGAACGUGUCACGUTT. All constructs were transfected into cells using Lipofectamine 2000 (Invitrogen), according to the manufacturer’s instructions.miR-26a-5p mimic and miR-26a-5p inhibitor were obtained from Beyotime (Beijing, China). Following incubation for 48 h, the transfected cells were har- vested and used for further experiments.

### RNA extraction and qRT-PCR

After 48 h post transfection, total RNA was extracted by using Trizol reagent (Invitrogen), respectively. RNA was resuspended in DEPC-treated H_2_O, and the concentration and purity were confirmed at 260 nm. For reverse transcription PCR, 10 mg of total RNA was converted to cDNA using cDNA Synthesis Kit (Bio-Rad), then PCR were performed. PCR was run at 25–30 cycles at 95 °C for 30 s, 56 °C for 30 s, and 72 °C for 1 min. U6 was used as an internal control. The relative expression levels were calculated by the 2^−ΔΔCt^ method. The primer sequences were designed by Beyotime (Beijing) and listed as follows: *SNHG6*, Forward primer: 5′-CCTACTGACAACATCGACGTTGAAG-3′; Reverse primer: 5′-GGAGAAAACGCTTAGCCATACAG-3′. miR- 26a-5p forward: 5′-GCGCGCGTAACAGTCTACAGC-3′ and reverse: 5′-GTCGTATCCAGTGCAGGGTCC-3′ *U6*: forward primer: 5′-CTCGCTTCGGCA GCA CA-3′ and reverse primer: 5′-AACGCTTCACGAATTTGCGT-3′. All reactions were performed at least three time.

### Western blot analysis

After 48 h post transfection, transfected cells were washed by ice-cold PBS and subjected to lysis in a RIPA buffer with 0.5% sodium dodecyl sulfate (SDS), 3% proteinase inhibitor cocktail (Sigma). Protein concentration was detected by BCA (bicinchoninic acid) protein assay. Then 15% sodium dodecyl sulfate polyacryl-amide gel electrophoresis gels (SDS-PAGE) and PVDF membranes (Millipore) were prepared to separate different protein. After blocking in 5% (w/v) non-fat dry milk for 1 h, PVDF membranes were incubated with primary antibodies against ULK1 (Cat. No.: ab203207), or Caspase 3 (Cat. No.: ab13585), or Activating transcription factor 3 (ATF3) (Cat. No.: ab207434) or GAPDH (Cat. No.: ab8245; abcam) overnight at 4 °C. Then membranes were incubated with horseradish peroxidase conjugated anti-mouse or rabbit secondary antibody (abcam) for 2 h. Finally, membranes were detected and analyzed using ECL Western Blotting Substrate (Thermo Fisher Scientific).

### Cell viability assay

3-(4,5-dimethylthiazol-2-yl)-2,5-diphenyltetrazolium bromide (MTT; Sigma) assay were used to detect cell viability. Cells transfected with SNHG6 siRNA or NC siRNA were seeded into 96 well plates and cultured overnight. Cells transfected nothing (Blank group) were used as control. Every 24 h interval, 20 ml of 5 mg/ml MTT was added into each well and cells were incubated for 2 h at 37 °C. Then 200 ml of DMSO (dimethyl sulfoxide; Sigma) were added to replace the medium. Optical density (OD) was evaluated at wavelength of 490 nm by measuring the absorbance. Cells treated with DMSO alone were performed to be a control. % viability survival = optical density of each group/optical density of DMSO group.

Each test was performed for five consecutive days and repeated in five wells. The experiments were repeated three times independently.

### Colony formation assay

Cells (500 cells per well) transfected with indicated vector were plated in 6-well plates and incubated at 37 °C. After 2 weeks, cells were fixed and stained with 0.1% crystal violet. Numbers of visible colonies were then counted manually after washing.

### Migration scratch assay

Transfected OS cells were placed on 12-well plates, then a cell‐scratch was made. Cell debris were washed by phosphate buffered saline (PBS) and cells were incubated at 37 °C for another 24 h. The rate of cell migration was determined by comparison of the images at 0 and 24 h after scratching. Each experiment was repeated three times.

### Trans-well migration assays

Boyden chamber with 8.0 μm pore (Corning) were used for this assay. 2 × 10^5^ transfected OS cells were seeded into the upper compartment in serum-free media. Media containing 10% FBS was placed in the lower chamber. Following 48 h incubation at 37 °C in 5% CO_2_, cells remaining in upper surface of the filter were wiped off with a cotton swab, while cells that migrated were fixed with methanol and stained with Giemsa (Sigma). Invasive cells were counted in five randomly light-microscopy fields. Each experiment was repeated in triple.

### Luciferase reporter assay

Wild-type (WT) fragments from *SNHG6* (*SNHG6*-Wt) containing relative predicted miR-26a-5p binding sites or mutated-type (MUT) *SNHG6* (*SNHG6*-Mut) sequence were synthesized and cloned downstream of the Dual-luciferase gene in the pGL3-basic vector (Promega, Madison, WI, USA). The cells in the logarithmic growth were inoculated into the 96-well plate and transfected using Lipofectamine 2000 (Invitrogen, NY, USA) when the cell density was about 70%. Dual-luciferase reporter plasmid containing *SNHG6*-Wt or *SNHG6*-Mut was co-transfected into HEK293T cells along miR-26a-5p mimics in parallel with an empty plasmid vector (miR-NC). After 24 h of transfection, relative luciferase activity was determined using a Dual luciferase assay kit (BioVision, San Francisco, CA, USA). The firefly luciferase activity was detected and normalized to Renilla activity. The experiment was repeated three times.

Similarly, WT or MUT *ULK1* sequence containing putative miR-26a-5p recognition sites was cloned downstream of the luciferase gene in the Dual-luciferase Vector to form the reporter vector *ULK1*-wild-type (*ULK1*-Wt) or *ULK1*-mutated-type (*ULK1*-Mut).

### Flow cytometry assay

For cell-cycle analysis, OS cells were seeded in 6 cm dishes at 5 × 10^4^/dish after transfection. After 48 h of cultivation, cells were washed and suspended in PBS containing 50 μg/ml RNase A (Sigma-Aldrich) and 50 μg/ml propidium iodide (PI; BD Biosciences, Erembodegem, Belgium) for 1 h in the dark. Fluorescence associated cellsorting (FACS) Verse™ flow cytometer (Becton–Dickinson, CA, USA) was used to count cell populations in each cell cycle phases and the results were analyzed with the ModFit (Verity Software House, Maine, USA) software.

For apoptosis analysis, cells (1 × 10^5^/well) were collected after 48 h of transfection and stained with both Annexin V–fluorescein isothiocyanate (FITC) and PI according to the manufacturer’s instructions (BD). Apoptosis was detected by FACSVerse™ flow cytometer.

### Statistical Analysis

Data were analyzed by GraphPad Prism 6.0 (GraphPad) and presented as mean ± standard deviation (SD). All *P* values were calculated using unpaired Student’s *t* test or ANOVA test with Tukey’s post hoc test, unless otherwise stated. Paired Student’s t-test (for parametric data) or Wilcoxon signed-rank test (for non-parametric data) were applied to analyze the differences of *SNHG6* expression levels between OS tissues and adjacent normal tissues. The Pearson’s Chi square were performed to determine the difference between *SNHG6* expression levels and clinic pathological factors. The overall survival probability was analyzed using Kaplan–Meier methods and evaluated using the log-rank test. *P* < 0.05 was considered as statistically significant. 3. Results

### SNHG6 was over-expressed in human osteosarcoma tissues

**Fig. 1:**
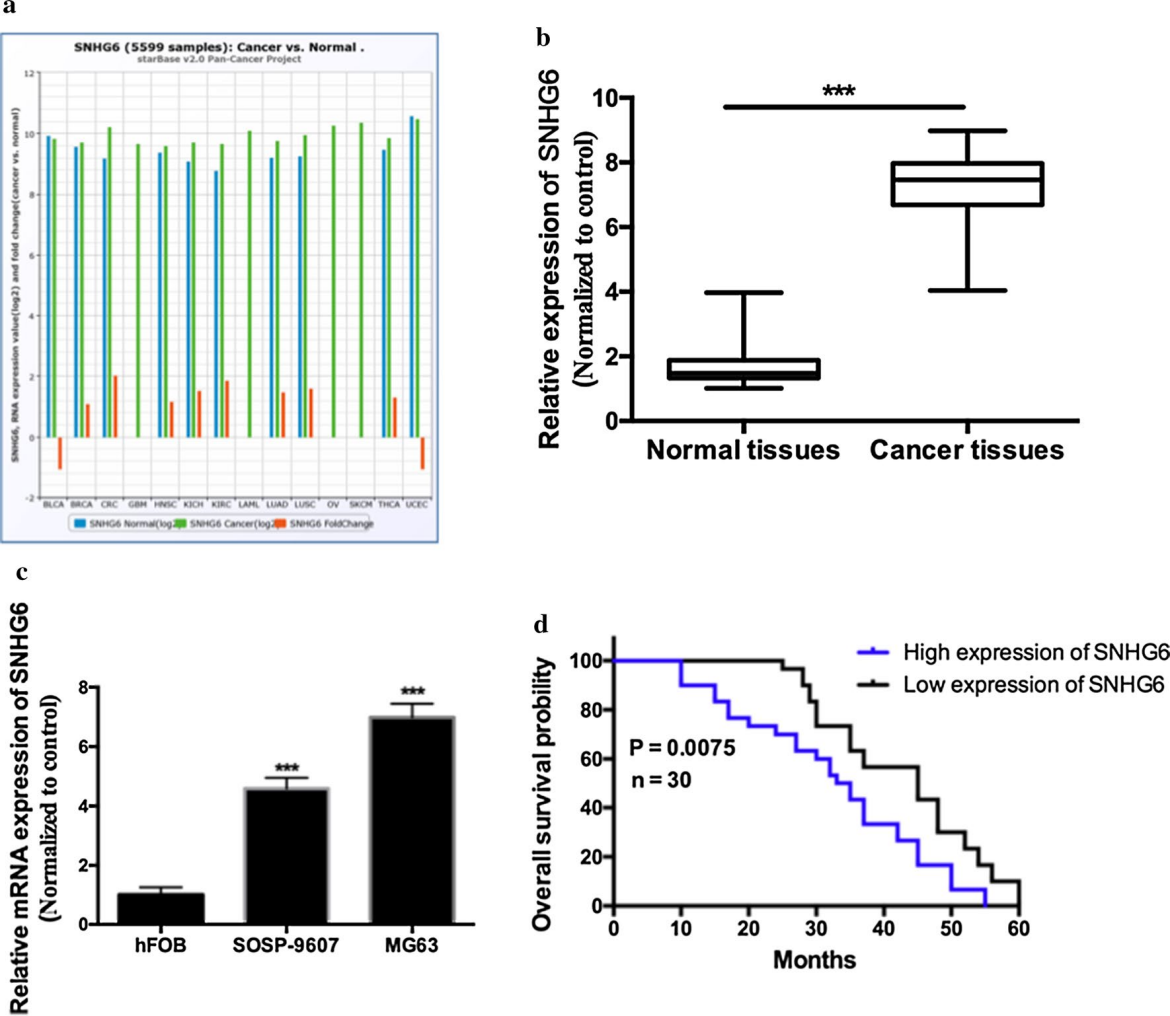
SNHG6 was over-expressed in human osteosarcoma. **a** SNHG6 expression of 14 cancer types from The Cancer Genome Atlas (TCGA) Data Portal from starBASE v2.0. **b** The relative expression of SNHG6 was examined by qRT-PCR in 30 pairs of OS tissues. **c** The relative expression of SNHG6 was examined by qRT-PCR in two OS cell lines (SOSP-9607, MG63) and one human normal osteoblastic cell line (hFOB). **d** Kaplan–Meier analysis revealed that OS patients with high level of SNHG6 suffered a poor overall survival. All samples were assayed in triplicate. *P < 0.05, ** P < 0.01, ***P < 0.001

To investigate whether *SNHG6* was correlated with the progression of OS patients, we explored *SNHG6* expression in The Cancer Genome Atlas (TCGA) Data Portal from starBASE v2.0 [15]. We found that *SNHG6* level was much higher in tumor tissues than in normal tissues among Pan-Cancer including 14 cancer types (breast cancer, clear cell kidney carcinoma, lung cancer, and so on) (Fig. 1a). Then we analyzed 30 pairs of human OS tissues samples (30 human OS tissues and 30 corresponding adjacent normal tissues) by using qRT-PCR. Comparing with matched adjacent normal tissues, *SNHG6* was significantly over-expressed in human OS cancer tissues by fourfold (Fig. 1b, *P *< 0.001). Besides, over expression of *SNHG6* level was also observed in two human OS cell lines (SOSP-9607 and MG63 cells) comparing with human hFOB cells (Fig. 1c, *P *< 0.001).

### High level *SNHG6* was correlated with poor overall survival in OS patients

To further understand the association of *SNHG6* in OS, we divided all OS tissue samples into 2 groups using median level of *SNHG6* as a cutoff value (high *SNHG6* expression group: *SNHG6* expression ratio > median level; low *SNHG6* expression group: *SNHG6* expression ratio ≤ median level). This finding demonstrated that higher *SNHG6* level was highly correlated with tumor invasion depth (Table 1, *P *= 0.001), lymph node metastasis (Table 1, *P *= 0.011) and TNM stage (Table 1, *P *= 0.001), while there was no significant correlation with gender, age and histological grade (*P *> 0.05). Additionally, Kaplan–Meier method analysis and log-rank test showed that patients with high level of *SNHG6* processed a shorter overall survival (Fig. 1d, *P *< 0.01). All these data implicated that *SNHG6* might implicated a poor prognosis.

### SNHG6 knockdown suppressed OS cell proliferation

**Fig. 2:**
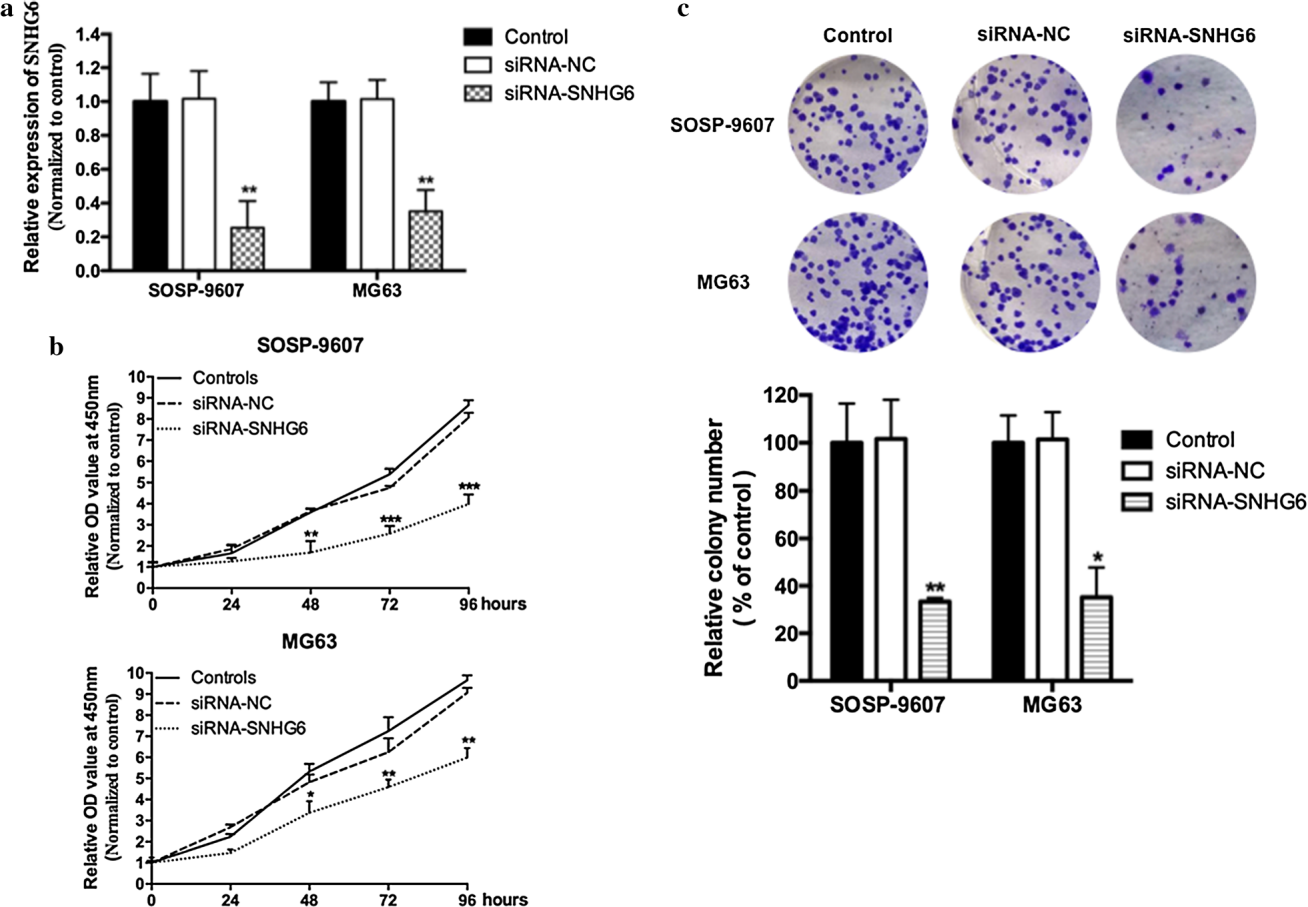
SNHG6 knockdown suppressed OS cell proliferation in vitro. **a** Relative expression of SNHG6 were decreased both in SOSP-9607 and MG63 cells after siRNA-SNHG6 transfection, comparing with OS cells transfected with siRNA-NC or non-transected cells, as assessed by qRT-PCR. **b** The proliferation ability of OS cells transfected with siRNA-SNHG6 were significantly decreased comparing with the cells transfected with siRNA-NC or non-transected cells. **c** The colony formation ability of OS cells transfected with siRNA-SNHG6 were significantly decreased comparing with the cells transfected with siRNA-NC or non-transected cells. All samples were assayed in triplicate. *P < 0.05, ** P < 0.01, ***P < 0.001

To clarify the function of *SNHG6* in OS, we reduced *SNHG6* level in SOSP-9607 and MG63 cells by transfecting siRNA specifically targeting *SNHG6* (siRNA-*SNHG6*). qRT-PCR assay was performed to confirmed the transfection effect (Fig. 2a, p < 0.01). Knockdown of SNHG6 decreased the SNHG6 expression levels by fourfold. MTT and colony formation assay were performed after transfection, and which suggested that knockdown of *SNHG6* markedly suppressed cell proliferation (Fig. 2b, *P *< 0.05) and colony formation (Fig. 2c, *P *< 0.05) in both OS cell lines.

### SNHG6 knockdown suppressed OS cell migration and invasion

**Fig. 3:**
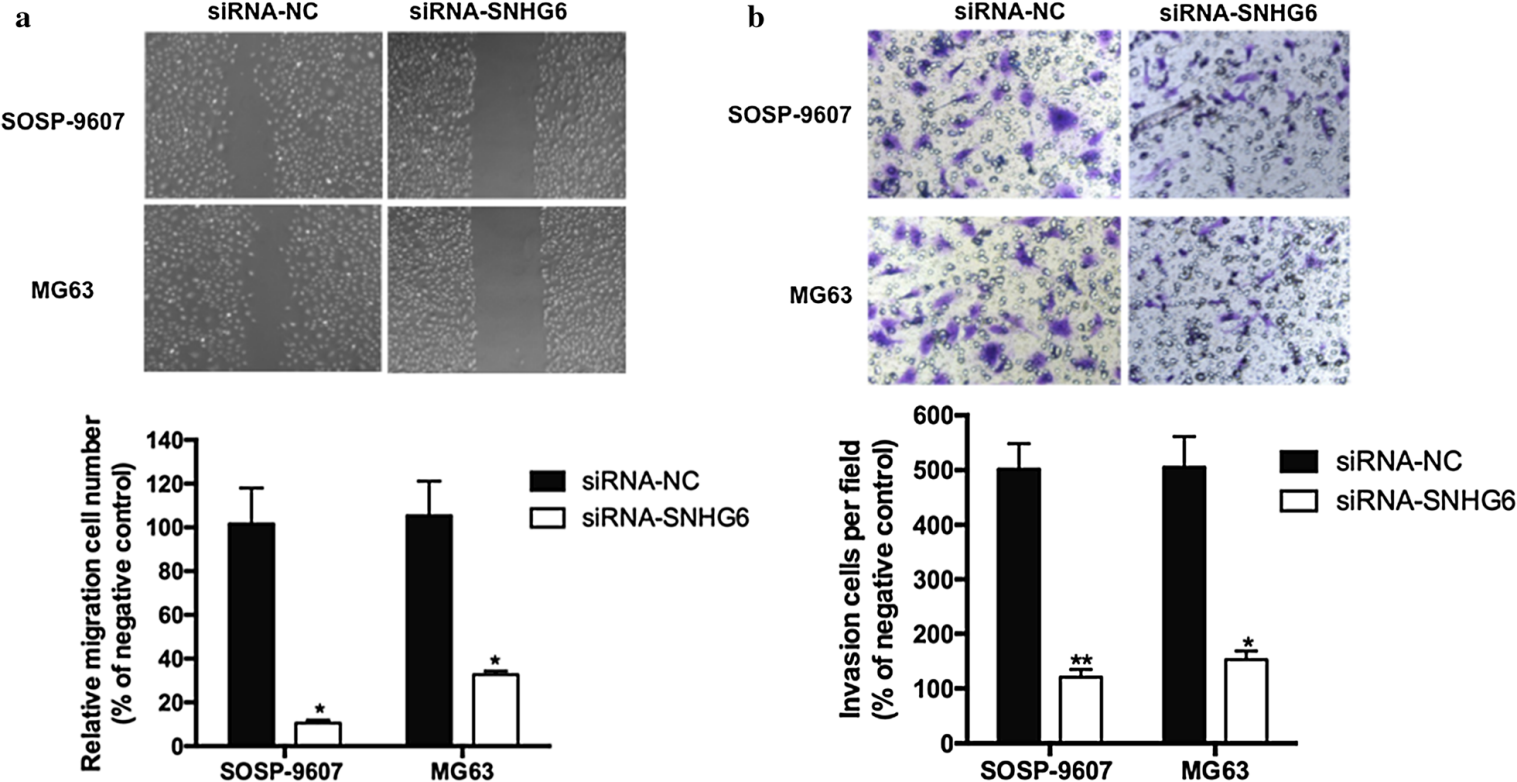
SNHG6 knockdown suppressed OS cell migration and invasion in vitro. **a** Cell migration of SOSP-9607 and MG63 cells transfected with the siRNA-SNHG6 or siRNA-NC were determined by wound healing assay, respectively. **b** Cell invasion of SOSP-9607 and MG63 cells transfected with siRNA-SNHG6 or siRNA-NC were determined by invasion chamber assay. All samples were assayed in triplicate. *P < 0.05, ** P < 0.01

Migration and invasion experiments were performed on SOSP-9607 and MG63 cells transfected with siRNA-*SNHG6* or siRNA-NC. *SNHG6* silencing significantly inhibited OS cells migration (Fig. 3a, *P* < 0.05) and invasion (Fig. 3b, *P* < 0.05), respectively.

### SNHG6 knockdown arrested cell cycle and cell apoptosis

**Fig. 4:**
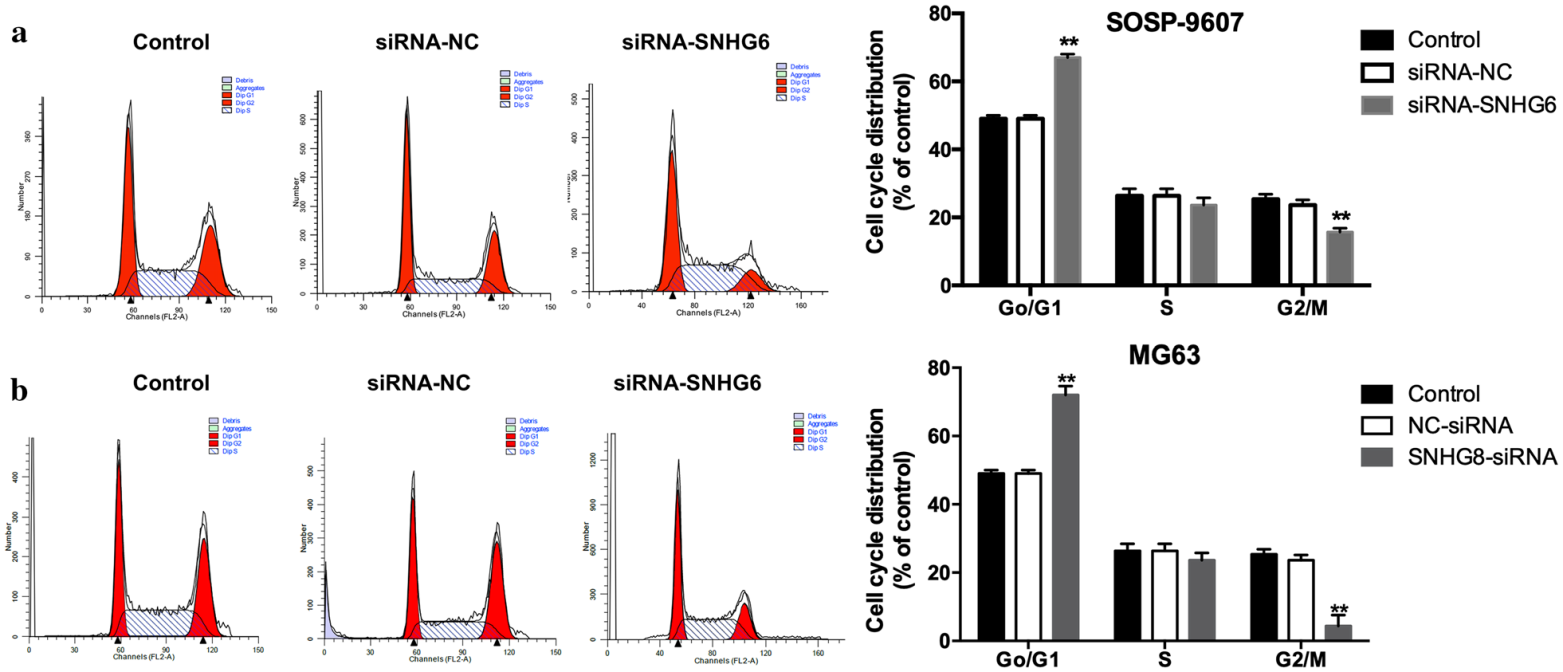
SNHG6 knockdown suppressed OS cell growth at G0/G1 phase. **a** Effect of SOSP-9607 transfected with siRNA-SNHG6 or siRNA-NC or none on cell cycle was detected by flow cytometry. **b** Effect of MG63 transfected with siRNA-SNHG6 or siRNA-NC or none on cell cycle was detected by flow cytometry. All samples were assayed in triplicate. ** P < 0.01

**Fig. 5:**
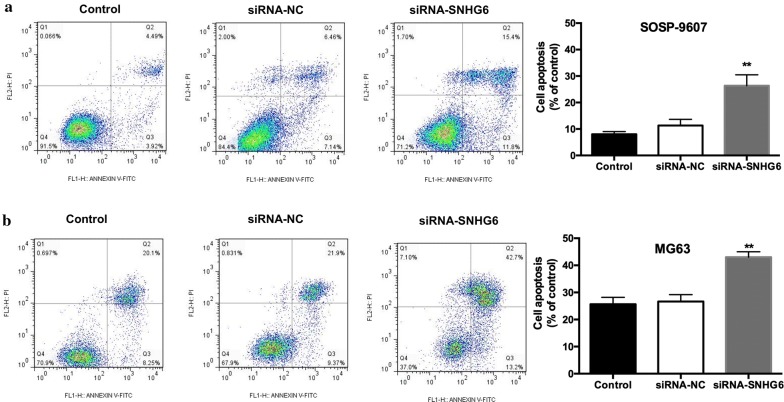
SNHG6 knockdown induced OS cell apoptosis. **a** Effect on cell apoptosis of SOSP-9607 transfected with siRNA-SNHG6 or siRNA-NC or none was detected by flow cytometry. **b** Effect on cell apoptosis of MG63 transfected with siRNA-SNHG6 or siRNA-NC or none was detected by flow cytometry. All samples were assayed in triplicate. **P < 0.01

Next, flow cytometric analysis were carried out to characterize the functions of *SNHG6* on OS cell cell-cycle or apoptosis, flow cytometric analysis was performed. Based on these results, a significant arrested at the G1/G0 phase were observed on OS cells transfected with siRNA-*SNHG6*, comparing with cells transfected with siRNA-NC (Fig. 4, *P *< 0.01). In the meantime, apoptosis cells increased significantly in OS cells transfected with siRNA-*SNHG6* (Fig. 5, *P *< 0.01). Thus, we confirmed that *SNHG6* silencing suppressed OS cell proliferation by inhibited cell cycle progression and induced cell apoptosis.

### miR-26a-5p was an inhibitory target of SNHG6

**Fig. 6:**
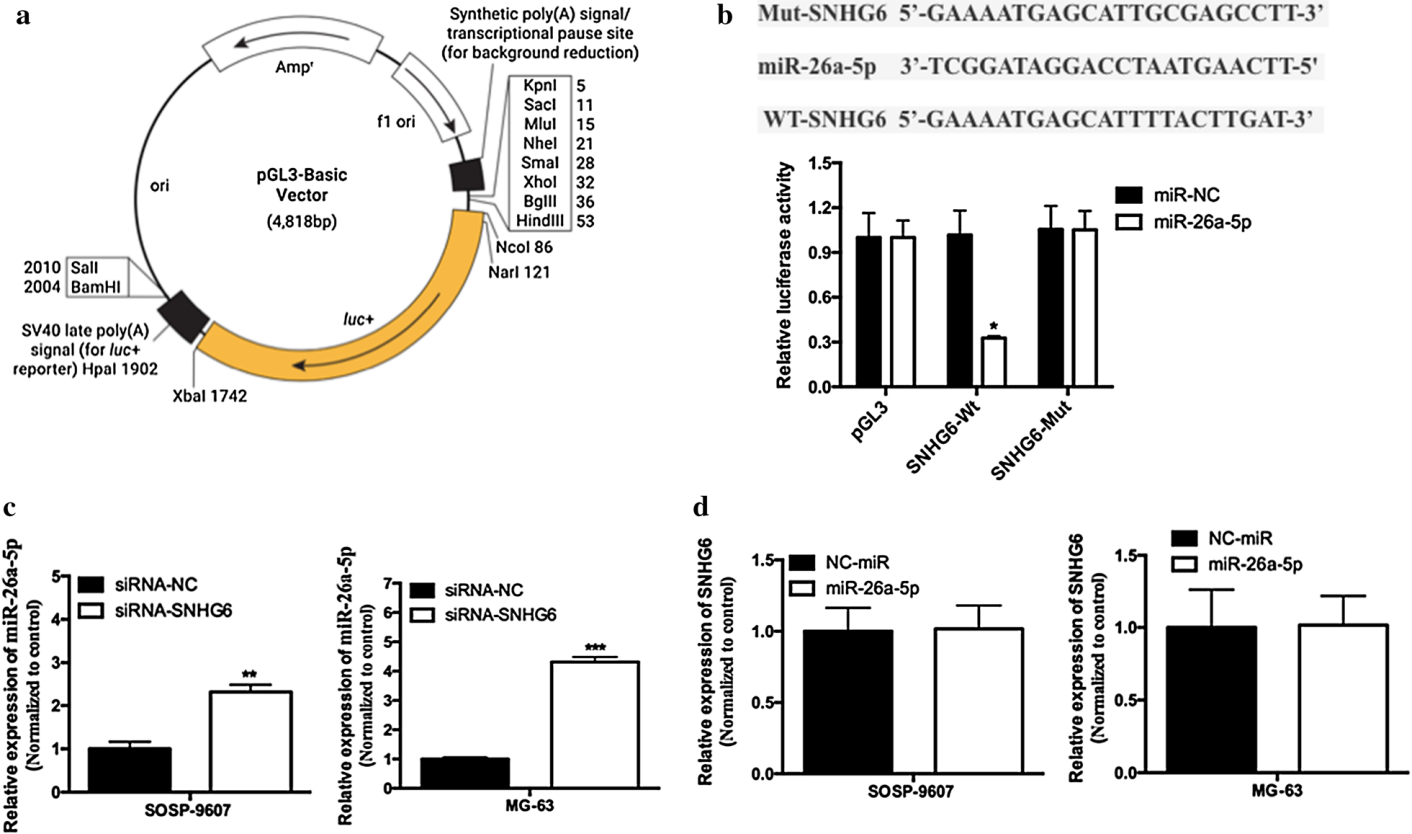
miR-26a-5p was an inhibitory target of SNHG6. **a** The pGL3 Basic Luciferase Reporter Vectors. **b** Sequence alignment of miR-26a-5p with the putative binding sites with in the wild-type regions of SNHG6. Dual-luciferase reporter assay showed that an obviously reduction of luciferase activity in 293T cells of SNHG6-Wt transfection, while no effect on the SNHG6-mut vector. **c** qRT-PCR analysis of miR-26a-5p expression in OS cells transfected with siRNA-SNHG6 or siRNA-NC. **d** qRT-PCR analysis of SNHG6 expression in OS cells transfected with miR-26a-5p or miR-NC. All samples were assayed in triplicate. *P < 0.05

Recently, various researches proved that lncRNAs mainly functioned as miRNA sponges by binding functional miRNAs and inhibited the transcription affection [16, 17]. To confirm whether *SNHG6* played the similar role in OS progression, we predicted the potential miRNAs associated with *SNHG6* by the online software starBase v2.0, 5 miRNAs, including miR-101-3p, miR-26a-5p, miR-1297, miR-26b-5p and miR-4465 were the potential targets of *SNHG6*, while none of them have been implicated in OS progression. To further confirmed this prediction, a Dual-luciferase reporter assay was used in 293T cells. The pGL3 Basic Luciferase Reporter Vectors was shown in Fig. 6a. The predicted target sites of miR-26a-5p binding to the 5′-UTR of *SNHG6* mRNA was showed in Fig. 6b. Co-transfection of miR-26a-5p and *SNHG6*-Wt but not the *SNHG6*-Mut induced a significantly inhibition on luciferase activity (Fig. 6a, P < 0.05), indicating miR-26a-5p might be a direct target of *SNHG6* in OS progression.

To further support this conclusion, qRT-PCR assay were performed. *SNHG6* silencing induced a significantly increased expression of miR-26a-5p (Fig. 6c, *P* < 0.01), while over-expressed of miR-26a-5p did not act on the expression of *SNHG6* (Fig. 6d, *P* > 0.05). In a summary, our research suggested that miR-26a-5p was a direct inhibitory target for *SNHG6* in OS progression.

### ULK1 was a target gene of miR-26a-5p and was regulated by SNHG6

**Fig. 7:**
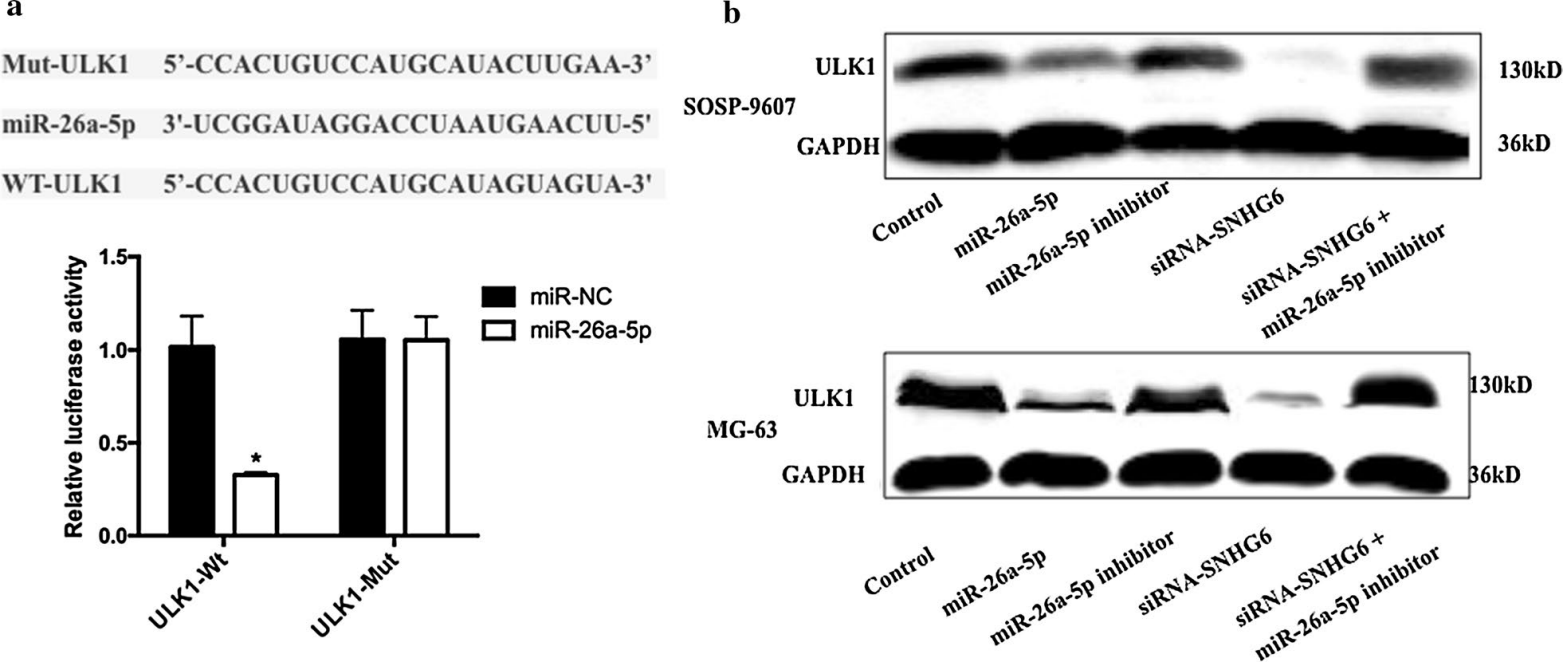
ULK1 was a target gene of miR-26a-5p and was regulated by SNHG6. **a** Dual-luciferase reporter assay revealed that miR-26a-5p inhibited ULK1-Wt luciferase activity, while it had no effect on ULK1-Mut luciferase activity in 293T cells. *P < 0.05. **b** Protein expression of ULK1 was determined by western blot in OS cells transfected with miR-26a-5p mimics, miR-26a-5p inhibitor, siRNA-SNHG6 or siRNA-SNHG6 plus smiR-26a-5p inhibitor. All samples were assayed in triplicate

ULK1, a serine/threonine protein kinase as the key component of autophagy pathway, came out as one of the target genes for miR-26a-5p [18]. To confirm the regulation of ULK1 by miR-26a-5p, Dual-luciferase reporter assay were performed in 293T cells. The predicted target sites of miR-26a-5p binding to the 5′-UTR of *ULK1* mRNA was showed in Fig. 7a. The luciferase activity was significantly inhibited while co-transfected with miR-26a-5p mimic and ULK1–3′UTR-wt plasmids (Fig. 7a, P < 0.05). To further confirm the regulation of ULK1 by miR-26a-5p, we examined the protein expression of ULK1 in OS cells transfected with miR-26a-5p, miR-26a-5p inhibitor, siRNA-*SNHG6* or both miR-26a-5p inhibitor and siRNA-*SNHG6*. Our results in Fig. 7b showed that increased expression of miR-26a-5p markedly increased the protein levels of ULK1 and this could been reversed by miR-26a-5p inhibitor. Knockdown of SNHG6 reduced the protein expression of ULK1, while knockdown of SNHG6 was no longer able to increase ULK1 protein level with miR-26a-5p inhibition. Collectively, these data might imply that ULK1 mRNA was the target of miR-26a-5p.

### SNHG6 regulated autophagy by targeting miR-26a-5p/ULK1

**Fig. 8:**
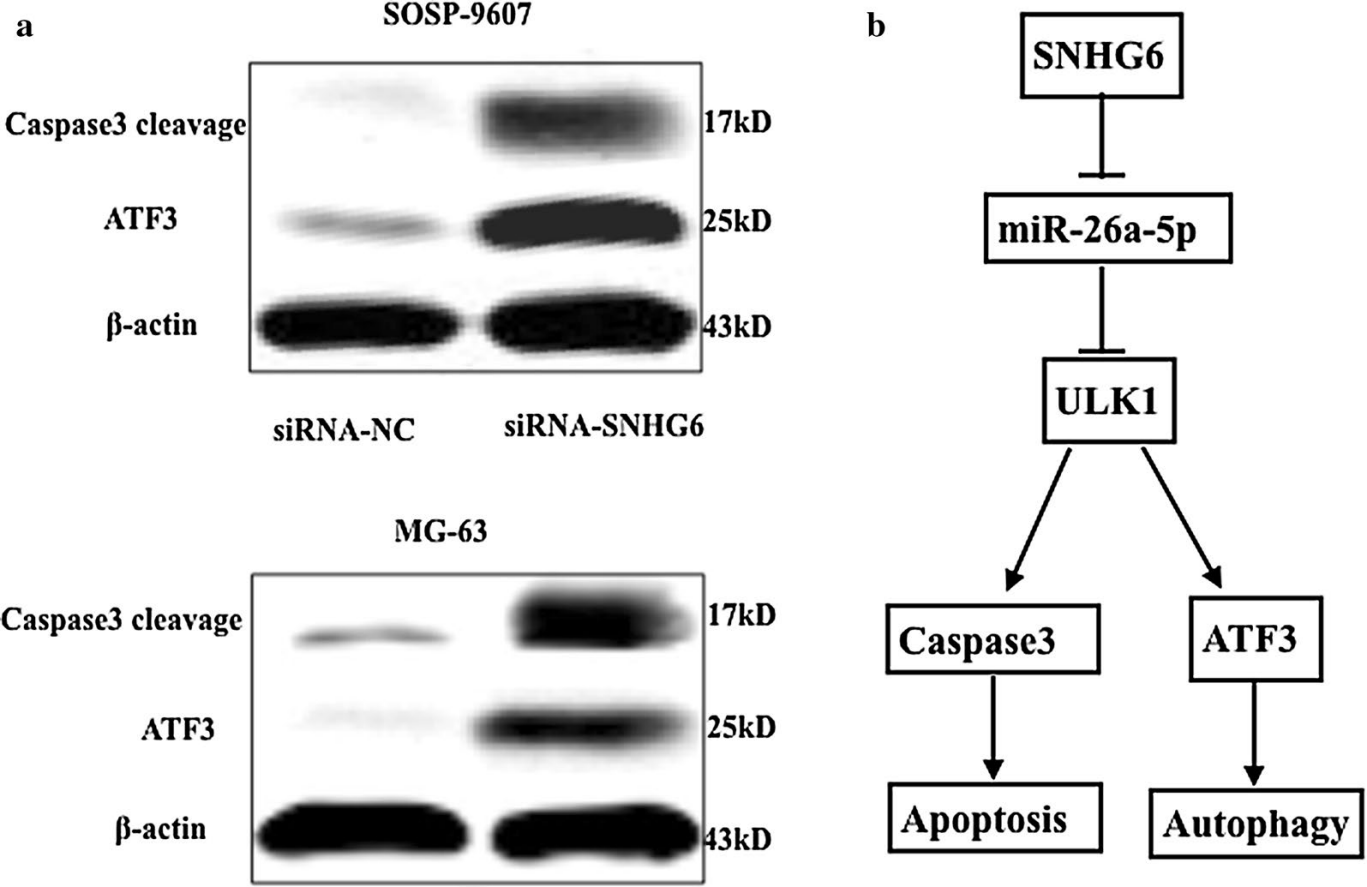
SNHG6 regulated autophagy by targeting miR-26a-5p/ULK1. **a** The cells were transfected with siRNA–siRNA or siRNA-NC, then the expression levels of cleaved-caspase3 and ATF3 were determined by Western blot analysis. β-actin was measured as loading control. **b** The schematic model of SNHG6 siRNA-induced cell death pathways, associated with autophagy and apoptosis. All samples were assayed in triplicate

Autophagy played opposed survival-supporting and death-promoting roles in cancer, which might be a tumor suppressor for preventing tumorigenesis, or act as an oncogene to promote cancer progression [19]. ULK1 and its complex has been reported as an essential role in autophagy induction [20, 21]. Activating transcription factor 3 (ATF3) was an autophagy-related protein, and caspase3 was known as the apoptotic executor, both were modulated by ULK1 in the downstream signals [22]. We then explored the relationship between SNHG6 and the ULK1 signaling pathway in OS cells by western blot analysis. As Fig. 8a shown, cells transfected with siRNA-SNHG6 had significantly decreased Caspase 3 exposure levels. Moreover, the expression of ATF3 in OS cells was significantly suppressed after SNHG6 siRNA transfection. All these results demonstrated that caspase3 and ATF3 were all involved in human OS cells death, which may be regulated by SNHG6/miR-26a-5p/ULK1 signaling pathway (Fig. 8b).

## Discussion

In the past decades, a substantial body of research have indicated the critical role of lncRNAs in carcinogenesis, included in osteosarcoma [23–25]. We focused on SNHG6 function because previous studies have demonstrated the potential oncogene function of SNHG6 to tumorigenesis. Recently, Chang et al. demonstrated that SNHG6 served as an oncogene by competitively binding miR-101-3p in human hepatocellular carcinoma [26]. Soon afterwards, Kai et al. demonstrated that SNHG6 promoted gastric cancer cells proliferation via miR-101-3p/ZEB1 regulation which performed at a post-transcriptional level [27]. However, more role of SNHG6 in human OS urgently need to be discovered.

Among this research, function and mechanism experiments were performed. Firstly, we found that SNHG6 expression was significantly increased in OS tissues comparing with corresponding normal tissues. The increased SNHG6 expression was determined to be significantly correlated with invasion depth and TNM stage, and positively correlated with poor prognosis of OS patients. Then loss-of-function assay was performed, and we found that silenced SNHG6 could decreased OS cell proliferation, migration and invasion. This was the first report of SNHG6 function in human OS, which may present a novel therapeutic strategy.

lncRNAs have been reported to regulate tumor development and progression by modulating different targets [28]. The most common regulation patten of lncRNAs was to be a direct targets of miRNAs as miRNA molecular sponge during the last few year, moreover, miRNA altered the expression of target mRNA [29, 30]. The lncRNAs-miRNA-mRNA axis seemed to be an extensive regulatory network in gene expression, and the extraordinary gene expression always indicated a disease progression, including cancer development. Likewise, in this investigation, SNHG6 silencing was attributed to its influence depending on its regulation on ULK1 by sponging suppress miR-26a-5p. Collectively, this is the first time that SNHG6 was identified to be a member of miR-26a-5p/ULK1 regulation axis in human OS development and progression, which probably offered an optional therapeutics against OS.

Autophagy was a process of cell self-digestion, which help to degrade and re-use those unwanted or dysfunctional components in cells, and turn out to help to kept cells in a state of dynamic equilibrium [31]. Autophagy acted as a double-edged sword in cancer. On the one hand, autophagy seemed to play a role as a suppressor by clearing mutant or misfolded proteins and alleviating cellular stress at the beginning of tumor development, [32] on the other hand, autophagy enabled tumor cells to survive under nutrient deficiency, hypoxia and other stresses, or to form a barrier to treatment with chemotherapeutic drugs. Thus, we speculated autophagy block might be beneficial to cancer therapy. However, most studies have focused on using common autophagy inhibitors, and most inhibiting targets were far downstream of the autophagy process [33]. ULK1, a serine/threonine protein kinase, which was one of the crucial factors for autophagy initiation and played a cardinal role in the regulation of autophagy [34]. Accumulating evidence suggestedULK1 act as a key initiator of autophagy. Zheng et al. firstly found that MiR-26a-5p regulate autophagic pathway in cardiac broblasts by targeting ULK1 [18]. Jin et al. demonstrated for the first time that miR-26a/b can promote apoptosis and sensitize hepatocellular carcinoma to chemotherapy via suppressing the expression of autophagy initiator ULK1 [35].

As the most common form of childhood and adolescent cancer, osteosarcoma suffered a rare survival rate for drug resistance. Inhibition of autophagy has been proved to increase the drug sensitivity of osteosarcoma cells in vivo and in vitro [36]. So we speculated whether SNHG6 promoted OS cells proliferation and reduced cell apoptosis through cell autophagy. Caspase-3 has been considered as crucial modulators of apoptosis. In our research, the expression level of Caspase-3 increased significantly following SNHG6 siRNA treatment, indicating that SNHG6 suppression promoted cell death by activating caspase-3-dependent apoptosis. Furthermore, SNHG6 siRNA significantly up-regulated the expressions of ATF3, implicating caspase3 and ATF3 were involved in SNHG6 siRNA induced cell death, which may be simultaneously regulated by miR-26a-5p/ULK1.

## Conclusions

In summary, we have newly identified the SNHG6/miR-26a-5p/ULK1 regulatory mechanism of autophagy in human osteosarcoma, and provided an optional choose for human OS treatment in the future.

## Abbreviations

OS: osteosarcoma
SNHG6: nucleolar RNA host gene 6
lncRNAs: long non-coding RNAs
ATF3: activating transcription factor 3
